# Supplementary material for: Extensive CArdioVAscular Characterization and Follow-Up of Patients Receiving Immune Checkpoint Inhibitors: A Prospective Multicenter Study
Source: Pharmaceuticals (Basel). 2023 Apr 20;16(4):625. doi: 10.3390/ph16040625 (PMC10142365; doi:10.3390/ph16040625)
Supplement: Supplementary file 1 [file pharmaceuticals-16-00625-s001.zip › pharmaceuticals-2326799-supplementary.pdf]

Supplementary Materials

Table S1. Schedule of activities.

Timepoint	Baseline	First 3 months	Month 3	Month 6	Month 12	Month 24
Informed consent	X					
Inclusion/exclusion criteria	X					
Medical history	X					
Prior/concomitant medication review	Continuous					
Physical examination†	X	X	X	X	X	X
Patient reported outcomes						
- Vital signs	Continuous					
- (S)AEs/irAEs	Continuous					
- Cardiovascular questionnaire	Continuous					
Cardiovascular analyses						
- 12-lead ECG	X	X‡	X	X	X	X
- 3D Echocardiography	X		X	X	X	X
- CT scan for calcium scoring	X				X	X
Laboratory parameters						
- Immune baseline blood analysis°	X					
- Immune follow-up blood analysis°		X	X	X	X	X
- Additional blood sample△	X		X	X	X	X
Follow-up						
- Survival status	Continuous					
- Treatment*	Continuous					

† All physical examinations will also include a neurological exam. ‡ An ECG will be taken by a qualified designee prior to each ICI cycle during the first three months of treatment. ° The specific laboratory values are listed in Supplementary Table S2 △ An additional blood sample (7.5 mL) will be taken and used for future determination of hs-TnI, hs-TnT and NT-proBNP. * If ICI treatment was discontinued, data was still collected regarding subsequent systemic therapies. Abbreviations: CT, computed tomography; hs-TnI, high-sensitivity troponin I; hs-TnT, high-sensitivity troponin T; irAE, immune-related adverse event; NT-proBNP, N-terminal pro-B-type natriuretic peptide; sAE, severe adverse event

Table S2. Laboratory parameters determined during immune baseline and immune follow-up blood samples analyses.

	Baseline (fasting)	Unit	Follow-up	Unit
Hematology	Red blood cell count		Red blood cell count	
	Hemoglobin	10^6 mm^3	Hemoglobin	10^6 mm^3
	Hematocrit	g/dL	Hematocrit	g/dL
	Mean corpuscular	%	Mean corpuscular volume	%
	volume	fL	Mean corpuscular	fL
	Mean corpuscular	pg	hemoglobin	pg
	hemoglobin	10^3 mm^3	White blood cell count	10^3 mm^3
	White blood cell count	10^3 mm^3	Platelet count	10^3 mm^3
	Platelet count	%	Neutrophil	%
	Neutrophil	%	Eosinophil	%
	Eosinophil	%	Basophil	%
	Basophil	%	Lymphocyte	%
	Lymphocyte	%	Monocyte	%
	Monocyte	10^3 mm^3	Absolute neutrophil	10^3 mm^3
	Absolute neutrophil	10^3 mm^3	Absolute eosinophil	10^3 mm^3
	Absolute eosinophil	10^3 mm^3	Absolute basophil	10^3 mm^3
	Absolute basophil	10^3 mm^3	Absolute lymphocyte	10^3 mm^3
	Absolute lymphocyte	10^3 mm^3	Absolute monocyte	10^3 mm^3
	Absolute monocyte			
Chemistry	Glucose **[fasting]**	mg/dL	Glucose [**non-fasting**]	mg/dL
	Urea	mg/dL	Urea	mg/dL
	Creatinine	mg/dL	Creatinine	mg/dL
	eGFR (CKD-EPI)	mL/min/1.73 m^2	eGFR (CKD-EPI)	mL/min/1.73
	Albumin	g/L	Albumin	m^2
	Bilirubin	mg/dL	Bilirubin	g/L
	Alkaline phosphatase	U/L	Alkaline phosphatase	mg/dL
	AST (= SGOT)	U/L	AST (= SGOT)	U/L
	ALT (= SGPT)	U/L	ALT (= SGPT)	U/L
	GGT	U/L	GGT	U/L
	Lipase	U/L	Lipase	U/L
	LDH (IFCC)	U/L	LDH (IFCC)	U/L
	Creatine kinase	U/L	Creatine kinase	U/L
	Troponin I or T	ng/L	Troponin I or T	U/L
	NT-proBNP	pg/mL		ng/L
Lipid panel	Triglycerides	mg/dL		
	Cholesterol	mg/dL		
	HDL-cholesterol	mg/dL		
	LDL-cholesterol	mg/dL		
Electrolytes & minerals	Sodium	mmol/L	Sodium	mmol/L
	Potassium	mmol/L	Potassium	mmol/L
	Chloride	mmol/L	Chloride	mmol/L
	Bicarbonate	mmol/L	Bicarbonate	mmol/L
	Calcium	mmol/L	Calcium	mmol/L
	Phosphorus	mmol/L	Phosphorus	mmol/L
	Magnesium	mmol/L	Magnesium	mmol/L
Hormonology	TSH	mIU/L		
	fT4	pmol/L		
	FSH	IU/L		
	LH	IU/L	TSH	mIU/L
	Estradiol	ng/L	fT4	pmol/L
	Testosterone	pg/mL	Cortisol	µg/dL
	ACTH	pg/mL		
	Cortisol	µg/dL		
Immunology and serology	CRP	mg/L		
	ANF (= ANA)	Positive or negative (if positive, ratio 1:XXX)		
	Anti-thyroglobulin Ab	IU/mL	CRP	mg/L
	HIV 1 & 2	Positive or negative		
	Hepatitis B sAg	Positive or negative		
	Hepatitis B sAb	IU/L		
	Hepatitis C Ab	Positive or negative		
